# Optimal set of grid size and angular increment for practical dose calculation using the dynamic conformal arc technique: a systematic evaluation of the dosimetric effects in lung stereotactic body radiation therapy

**DOI:** 10.1186/1748-717X-9-5

**Published:** 2014-01-04

**Authors:** Ji-Yeon Park, Siyong Kim, Hae-Jin Park, Jeong-Woo Lee, Yeon-Sil Kim, Tae-Suk Suh

**Affiliations:** 1 Department of Biomedical Engineering, The Catholic University of Korea Songeui Campus, Banpo4-dong, Seocho-gu, Seoul 137-701, Korea; 2 Research Institute of Biomedical Engineering, The Catholic University of Korea Songeui Campus, Banpo4-dong, Seocho-gu, Seoul 137-701, Korea; 3 Department of Radiation Oncology, Virginia Commonwealth University, Richmond, VA 23298, USA; 4 Department Radiation Oncology, Ajou University School of Medicine, Suwon 443-721, Korea; 5 Department of Radiation Oncology, Konkuk University Medical Center, Seoul 143-729, Korea; 6 Department of Radiation Oncology, Seoul St. Mary’s Hospital, Seoul 137-701, Korea

**Keywords:** Dynamic conformal arc therapy, Grid size, Angular increment, Lung, Stereotactic body radiation therapy

## Abstract

**Purpose:**

To recommend the optimal plan parameter set of grid size and angular increment for dose calculations in treatment planning for lung stereotactic body radiation therapy (SBRT) using dynamic conformal arc therapy (DCAT) considering both accuracy and computational efficiency.

**Materials and methods:**

Dose variations with varying grid sizes (2, 3, and 4 mm) and angular increments (2°, 4°, 6°, and 10°) were analyzed in a thorax phantom for 3 spherical target volumes and in 9 patient cases. A 2-mm grid size and 2° angular increment are assumed sufficient to serve as reference values. The dosimetric effect was evaluated using dose–volume histograms, monitor units (MUs), and dose to organs at risk (OARs) for a definite volume corresponding to the dose–volume constraint in lung SBRT. The times required for dose calculations using each parameter set were compared for clinical practicality.

**Results:**

Larger grid sizes caused a dose increase to the structures and required higher MUs to achieve the target coverage. The discrete beam arrangements at each angular increment led to over- and under-estimated OARs doses due to the undulating dose distribution. When a 2° angular increment was used in both studies, a 4-mm grid size changed the dose variation by up to 3–4% (50 cGy) for the heart and the spinal cord, while a 3-mm grid size produced a dose difference of <1% (12 cGy) in all tested OARs. When a 3-mm grid size was employed, angular increments of 6° and 10° caused maximum dose variations of 3% (23 cGy) and 10% (61 cGy) in the spinal cord, respectively, while a 4° increment resulted in a dose difference of <1% (8 cGy) in all cases except for that of one patient. The 3-mm grid size and 4° angular increment enabled a 78% savings in computation time without making any critical sacrifices to dose accuracy.

**Conclusions:**

A parameter set with a 3-mm grid size and a 4° angular increment is found to be appropriate for predicting patient dose distributions with a dose difference below 1% while reducing the computation time by more than half for lung SBRT using DCAT.

## Background

Stereotactic body radiation therapy (SBRT) has been employed to increase the local tumor control of lung and liver cancer treatments [1,2]. Various techniques related to the treatment planning, the beam delivery, and the imaging have been developed to achieve a more accurate and conformal dose distribution [3-6]. To effectively deliver the conformal dose distribution to the planning target volume (PTV) while sparing organs at risk (OARs), an arc track-based beam irradiation technique and a multi-leaf collimator (MLC) for adjusting the field shape are used to implement dynamic conformal arc therapy (DCAT) and volumetric modulated arc therapy (VMAT).

DCAT can provide conformal dose distributions by delivering doses with an MLC dynamically conforming its shape to the beam’s-eye-view projections of the PTV with relatively fast dose computation [3,7-9], while VMAT delivers the optimized dose distribution using a small beamlet-based intensity modulation by a combination of several separated MLC segments per beam [9]. Even though DCAT does not use intensity-modulated beams, it can still be used to satisfy the conformity requirements of planned doses [9,10]. However, contrary to the continuous beam delivery with a rotating gantry in the DCAT implementation, a grid-based discretized dose calculation for a number of beams with dose variation depending on their angular increment can cause systematic dose errors.

Although a smaller grid size can yield a more accurate and conformal dose calculation, particularly in regions of high dose gradient, dose calculations using a finer calculation grid size require a longer computational time [11,12]. The number of control points determined by the angular increment also influences the calculation accuracy and time. It is critical to determine plan parameters to balance the dose calculation accuracy and the computational time efficiency in order to maximize the quality of the plan while minimizing the planning time [11,13-15] for efficient clinical application of DCAT. When several dose computations are needed to determine a more conformal dose distribution by verifying the variation in dose distributions during a trial-and-error-based forward plan optimization process, a compromise between accuracy and efficiency is inevitable in DCAT planning [16]. An evaluation of the dosimetric impact of planning parameters would serve as an important step toward creating a SBRT plan using an appropriate grid size and angular increment.

The purpose of this study was to determine the optimal plan parameter set of values for the dose calculation grid size and angular increment for lung SBRT using the DCAT technique that will provide clinically acceptable dose accuracy and efficient computational time. Dose variations and computation times with different grid sizes and angular increments were analyzed for spherical tumors in thorax phantom image sets and patient computed tomography (CT) images. A systematic evaluation of the dose variations and the calculation time would provide a guideline for selecting appropriate planning parameters and estimating dose errors for DCAT.

## Materials and methods

### Planning system and dose calculation

Dose distributions for lung SBRT plans using DCAT were calculated by the analytical anisotropic algorithm (AAA) (v. 8.6.15, Varian Medical Systems, Palo Alto, CA) in an Eclipse planning system (v. 8.6.17, Varian Medical Systems). The dose distributions were calculated in the planning system with 24 GB of RAM and a dual-core processor running under the Microsoft Windows® 64-bit operating system. We adopted a commonly used dose-fractionation scheme of 48 Gy in 4 fractions for lung SBRT [1] to estimate the delivered doses using CT image sets of 3-mm thickness for the phantom and patient studies.

### Phantom study

**Figure 1:**
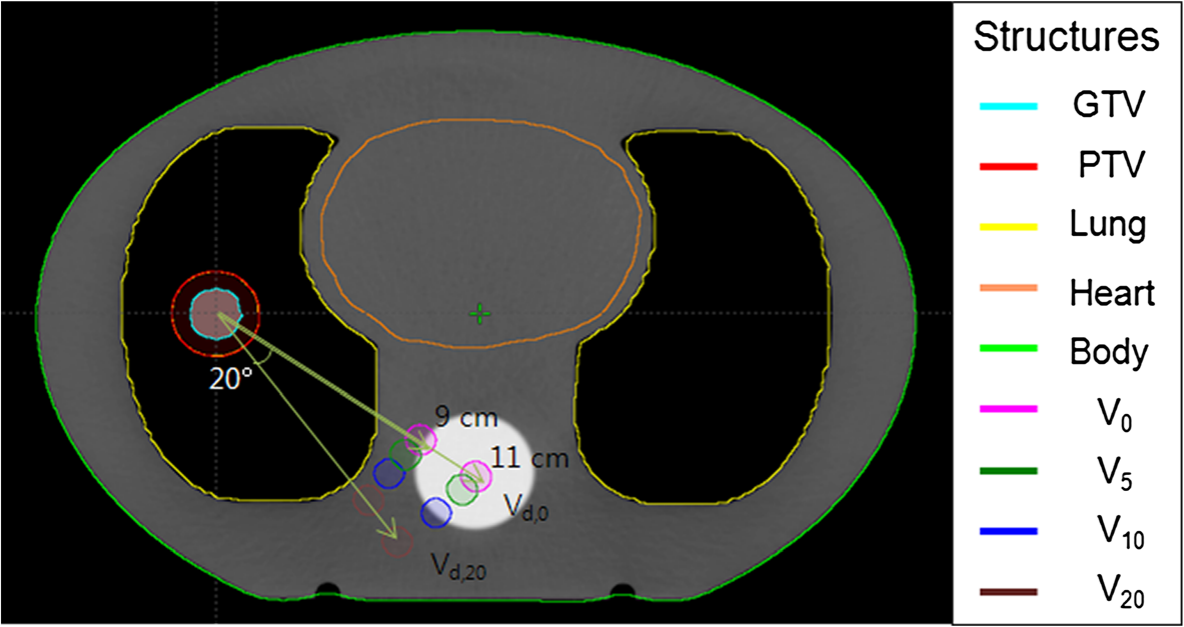
**A phantom computed tomography (CT) image and delineated planning target volume (PTV) and organs at risk (OARs).** The clinical target volume (CTV) overridden with 75 Hounsfield Unit value, the PTV created by adding a 5 mm-margin to the CTV, and the OARs (including the lung, the heart, and the spinal cord) were contoured on the phantom CT image sets. A set of 8 simulated spinal cord volumes were placed in different distances with 4 located 9 cm from the isocenter, and the other 4 at 11 cm from it. When a line connecting the isocenter and the original spinal cord in the phantom was chosen as the 0° azimuthal angle, and the other simulated spinal cords were positioned at 0°, 5°, 10°, and 20° in the clockwise direction. Each volume is denoted as *V*_ds,Θ_, where *d*_s_ is the distance from the isocenter to each spinal cord, and *Θ* is the azimuthal angle.

To estimate the dose variation in the tumor volumes and OARs with variable plan parameter sets (i.e., combinations of a variable grid size and an angular increment), 3 spherical clinical target volumes (CTVs) with diameters of 1, 2, and 3 cm and the OARs including the spinal cord, the lung, and the heart were delineated in a thorax phantom (002LFC, CIRS, Inc., Norfolk, VA). After averaging the Hounsfield unit (HU) values of the CTVs defined in selected patients for this study, the average value of 75 HU was overridden to the CTVs in the phantom to simulate dosimetric effects in the cases of individual patients (Figure 1). To define PTVs with diameters of 2 cm (*S*_2_), 3 cm (*S*_3_), and 4 cm (*S*_4_), a 5-mm margin was added to each CTV with diameters of 1 cm, 2 cm, and 3 cm, respectively, in consideration of organ motion and set-up misalignment.

When the numerical beams at every angular increment are irradiated to cover the PTV with the prescribed dose, a uniform dose distribution is created near the isocenter. As the distance between the axes of adjacent beams increases at regions far from the isocenter, the irradiated and unirradiated regions are sectionalized according to the angular increment. Because OARs of small volume can be placed on or off the beam path according to the distance from the field axis to the OAR, the predicted OAR doses are varied according to the angular position of the OAR with respect to the isocenter even in the same plan. To evaluate whether the plan parameter sets can provide an acceptable calculation accuracy of the OAR doses for the diverse cases encountered in our study, we delineated hypothetical OARs at different distances and angular positions from the isocenter. A set of 8 different spinal cords having a relatively small volume was arranged, as shown in Figure 1, with 4 of them at a distance (*d*_*s*_) of 9 cm from the isocenter in the axial plane and the other 4 at a distance of 11 cm from the isocenter. Four hypothetical spinal cords at the same distance were separated (clockwise) by 5°, 10°, and 20° with respect to the one chosen as the 0° azimuthal angle. These simulated spinal cords were denoted as *V*_*ds*,Θ_ where *d*_*s*_ is the distance, and Θ is the azimuthal angle. For example, *V*_9,20_ indicates the (hypothetical) spinal cord volume located at a 9-cm distance and a 20° azimuthal angle.

### Patient study

Dose variations as a function of variable grid size and angular increment were also evaluated to determine the appropriate plan parameter sets in patient cases having different volumes and positions of structures. Contrary to the phantom study in which all the PTV volumes were spherical, relatively irregularly shaped PTVs were chosen for the patient study. A total of 9 lung SBRT patient data sets were retrospectively selected. As the patients for lung SBRT undergo four-dimensional (4D) CT scanning to measure the motion range of tumors at our institution, the internal target volume (ITV) was defined using 4D CT images. The PTV was created by adding a 0.5-cm margin in the axial plane and 1.0 cm-margin in the longitudinal plane to the ITV following the referenced SBRT protocol [1]. For all patients, the PTV was smaller than an equivalent diameter (*d*_equi_) of 5.5 cm and located at more than 2 cm from the bronchial tree [1], where *d*_equi_ was defined as the diameter of a sphere having the same volume as the PTV.

Because the dosimetric effects on OARs distant from the isocenter can be varied by changing the field size for PTV, the patients were divided into 3 groups based on the *d*_equi_ of the PTV: small (3 cm ≤ *d*_equi_ < 3.5 cm) (G_1_), medium (3.5 cm ≤ *d*_equi_ < 4.5 cm) (G_2_), and large (4.5 cm ≤ *d*_equi_ ≤ 5.5 cm) (G_3_). In each group, 3 patients with spinal cords proximal to the isocenter (*d*_*s*_ < 6 cm), at an intermediate position (6 cm ≤ *d*_*s*_ < 10 cm), and distant (10 cm ≤ *d*_*s*_) from the isocenter were included to evaluate the dose variation according to the plan parameter sets.

### Plan parameters and evaluation

To determine the optimal grid size and angular increment to achieve a fast dose calculation without compromising dose calculation accuracy, dose distributions were calculated using grid sizes of 2, 3, and 4 mm and angular increments of 2°, 4°, 6°, and 10° in both the phantom and patient studies. The four angular intervals were selected based on plan parameters for linac-based radiosurgery and arc therapy [12,17]. Plan optimization that allowed coverage of 95% of the PTV with the prescribed dose in the same calculation volume was carried out.

The dosimetric effects of each plan parameter were evaluated using the dose–volume histograms (DVHs) and the variation in the OAR doses. Considering the dose constraints in the SBRT protocol [1], the OAR doses were evaluated at the same volume as the “Max Point Dose” for serial tissue and the “Critical Volume Dose Max” for parallel tissue. Because most of the primary OARs in lung SBRT are classified as serial tissues except for the normal lung in the SBRT protocol, the corresponding normal lung dose at a volume of 1000 cc and the maximum dose of other OARs were analyzed according to the dose constraint in the four-fraction scheme as shown in Table 1[1]. We summarized the relative variation in monitor units (MUs) and structure doses by normalizing them with respect to values in a reference plan using a 2-mm grid size and a 2° angular increment. The reference MU and doses were presented as absolute values. The computation times were compared to evaluate the time efficiency for each of the dose calculations for an SBRT plan.

**Table 1:** **Dose constraints for organs at risk in lung stereotactic body radiation therapy**[1]**, when the dose scheme of 12 Gy in 4 fractions is used**

**Type**	**Organs**	**Max point dose**	
Serial tissue	Spinal cord	26 Gy	
	Esophagus	30 Gy	
	Heart	34 Gy	
Parallel tissue	Organs	Critical volume	Critical volume dose max
	Lung (right + left)	1000 cc	12.4 Gy

A dose calculation uncertainty of less than 2% has been recommended in inhomogeneous media to ensure that the total uncertainty in dose delivery to patients is less than 5% [18-20]. When the doses predicted by AAA were delivered to a lung phantom, the total dose uncertainty has been estimated to be approximately 4% [20,21]. If we consider an uncertainty of 2.5%, caused by patient set-up and organ motion during treatment, an additional dose calculation uncertainty due to selecting the large grid size and angular increment should be less than 1.7% to meet the criteria of the total dose uncertainty. A plan parameter set which yields a dose distribution with a dose difference less than 1% and reduced calculation time could be judged as suitable for dose calculations in DCAT, because other larger errors can come into play in dose delivery to inhomogeneous materials.

## Results

### Phantom study

The PTV and OARs showed dose variations according to the plan parameter sets, the structure’s position with respect to the isocenter, and the field sizes. Quantitative changes in MUs and the dose variation in the PTV and OARs are summarized in Table 2.

**Table 2:** **Monitor units (MUs) and structure doses in the form of a ratio normalized to a reference value from the case of a 2-mm grid size and 2° angular increment, when different plan parameter sets (grid sizes of 2 mm, 3 mm, and 4 mm, and angular increments of 2°, 4°, 6°, and 10°) were used in the thorax phantom for the cases of spherical targets [S**_**2 **_**(2-cm diameter), S**_**3 **_**(3-cm diameter), and S**_**4 **_**(4-cm diameter)]**

	**Grid size (mm)**	**Angular increment (°)**	**MU**	**Structures**										
				**PTV (D**_**mean**_**)**	**Heart (D**_**max**_**)**	**Normal lung (D**_**1000cc**_**)**	**Spinal cord (D**_**max**_**)**							
							**V**_**9,0**_	**V**_**9,5**_	**V**_**9,10**_	**V**_**9,20**_	**V**_**11,0**_	**V**_**11,5**_	**V**_**11,10**_	**V**_**11,20**_
S_2_	2	2	1.00 (1638)^a^	1.00 (50.37)^b^	1.00 (13.60)	1.00 (7.40)	1.00 (6.92)	1.00 (7.19)	1.00 (7.44)	1.00 (7.48)	1.00 (5.57)	1.00 (5.77)	1.00 (6.09)	1.00 (6.34)
		4	0.99	1.00	1.00	1.12	1.00	1.00	1.00	1.00	1.00	1.01	0.99	1.00
		6	0.99	1.00	1.00	1.12	1.01	1.01	1.01	1.02	1.03	1.00	1.00	1.02
		10	0.99	1.00	1.00	1.11	1.05	1.07	1.00	1.04	1.09	1.06	1.07	1.06
	3	2	1.01	1.00	1.01	1.00	1.01	1.00	1.01	1.01	1.01	1.01	1.00	1.00
		4	1.00	1.00	1.01	1.12	1.00	1.01	1.00	1.00	1.01	1.01	1.00	1.00
		6	1.00	1.00	1.00	1.12	1.01	1.01	1.01	1.02	1.03	1.01	1.00	1.01
		10	1.00	1.00	1.00	1.12	1.05	1.04	1.00	1.03	1.10	1.06	1.07	1.06
	4	2	1.03	1.01	1.04	1.05	1.02	1.03	1.02	1.03	1.03	1.03	1.03	1.02
		4	1.01	1.00	1.02	1.16	1.01	1.01	1.01	1.01	1.02	1.01	1.01	1.00
		6	1.01	1.00	1.03	1.16	1.01	1.03	1.01	1.01	1.02	1.01	1.01	1.02
		10	1.01	1.00	1.04	1.16	1.05	1.06	1.01	1.05	1.10	1.07	1.08	1.07
S_3_	2	2	1.00 (1518)	1.00 (46.41)	1.00 (18.09)	1.00 (1.76)	1.00 (9.18)	1.00 (9.65)	1.00 (10.03)	1.00 (10.10)	1.00 (7.31)	1.00 (7.61)	1.00 (8.12)	1.00 (8.45)
		4	1.00	1.00	1.00	1.00	1.00	1.00	1.00	1.00	1.01	1.01	1.00	1.00
		6	1.00	1.00	1.00	0.99	1.01	1.00	1.01	1.01	1.01	1.02	1.01	0.99
		10	1.00	1.00	1.00	0.99	1.01	1.00	1.02	0.99	1.03	1.09	1.01	1.05
	3	2	1.00	1.00	1.01	1.01	1.00	1.00	1.00	1.00	1.00	1.00	1.00	1.00
		4	1.00	1.00	1.00	1.01	1.00	1.00	1.00	1.00	1.01	1.01	1.01	1.00
		6	1.00	1.00	1.00	0.99	1.01	1.00	1.00	1.01	1.01	1.02	1.01	0.99
		10	1.00	1.00	1.00	1.00	1.00	1.00	1.00	0.99	1.03	1.09	1.01	1.04
	4	2	1.01	1.00	1.01	1.02	1.00	1.00	1.00	1.00	1.00	1.00	1.00	1.00
		4	1.01	1.00	1.02	1.03	1.01	1.01	1.00	1.00	1.00	1.00	1.00	1.00
		6	1.01	1.00	1.01	1.02	1.01	1.00	1.01	1.01	1.00	1.00	1.01	1.00
		10	1.01	1.00	1.01	1.02	1.00	1.00	1.01	0.99	1.02	1.08	1.01	1.04
S_4_	2	2	1.00 (1454)	1.00 (45.00)	1.00 (23.03)	1.00 (3.13)	1.00 (11.42)	1.00 (11.96)	1.00 (12.37)	1.00 (12.50)	1.00 (9.00)	1.00 (9.37)	1.00 (9.98)	1.00 (10.38)
		4	1.00	1.00	1.00	1.00	1.00	1.00	1.00	1.00	1.01	1.01	1.00	1.00
		6	1.00	1.00	1.00	1.00	1.00	1.00	1.00	1.00	1.03	1.00	0.99	1.01
		10	1.00	1.00	1.00	1.01	1.02	0.99	1.01	1.02	0.98	1.04	0.99	0.99
	3	2	1.00	1.00	1.00	1.00	1.00	1.00	1.00	1.00	1.00	1.00	1.00	1.00
		4	1.00	1.00	1.00	1.00	1.00	1.00	1.00	1.00	1.00	1.00	1.00	1.00
		6	1.00	1.00	1.01	1.00	1.00	1.00	1.00	1.00	1.01	0.99	0.99	1.01
		10	1.00	1.00	1.00	1.00	1.01	0.99	1.01	1.02	0.98	1.03	0.99	0.99
	4	2	1.00	1.00	1.01	1.01	1.00	1.00	1.00	1.00	1.00	1.00	1.00	1.00
		4	1.00	1.00	1.01	1.01	1.00	1.00	1.00	1.00	1.00	1.00	1.00	1.00
		6	1.00	1.00	1.01	1.01	1.00	1.00	1.00	1.00	1.00	1.00	1.00	1.00
		10	1.00	1.00	1.01	1.01	1.00	1.00	1.00	1.00	1.00	1.00	1.00	1.00

a) The reference MU value and b) the reference dose (Gy) in each case were presented in parentheses.

**Figure 2:**
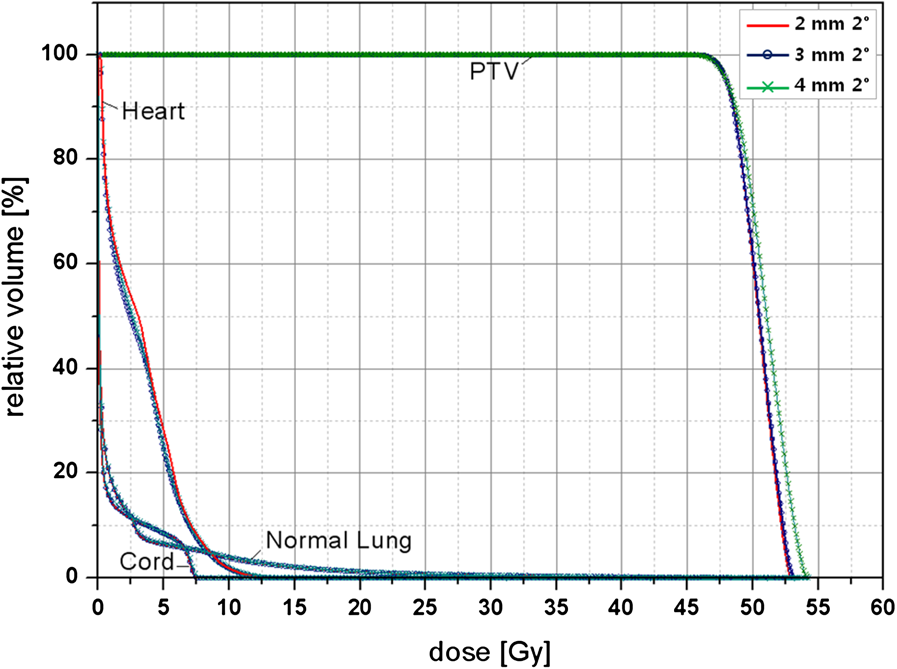
**Dose–volume histograms for grid sizes of 2 mm, 3 mm, and 4 mm in the plan for *S***_**2**_**.** Each plan was optimized to fully cover 95% of the planning target volume with the prescribed dose.

Dose differences due to the effects of grid size and angular increment became more obvious in the DCAT plan for the smaller PTV of *S*_2_. When the dose distributions calculated for different grid sizes and an angular increment of 2° were compared in *S*_2_, the grid size of 4 mm required higher values of the MU and showed higher *D*_mean_ of the PTV up to a maximum increase of 3% for the MU (> 50.0) and a 1% increase for the *D*_mean_ (60.8 cGy) than that of the 2-mm grid size, respectively. The maximum dose of the spinal cord and *D*_1000cc_ of the lung increased by 3% (19.6 cGy) and 5% (4.0 cGy), when the large grid size of 4 mm and 10° angular increment were used. The grid size of 3 mm led to a dose difference of less than 1% in the MU (< 14), PTV (< 6.4 cGy), spinal cord (< 5.5 cGy), and heart (< 8.6 cGy). Although the dose difference due to the grid size effect was difficult to discern in the DVH of the OARs, we could observe that the DVH lines of the PTV shifted to the right (i.e., toward the higher dose range) for all cases tested, as the grid size increased (Figure 2).

When the same angular increment was used for dose calculations with smaller grid sizes (i.e., a 4-mm grid with a 10° increment vs. a 2-mm grid with a 10° increment), the largest grid size caused the maximum dose difference of 4% (41.7 cGy) in the spinal cord in *S*_4_ and 4% (47.6 cGy) in the heart in *S*_2_. Even though the dose calculation using a 3-mm grid size also caused an increase in the MUs and average dose (*D*_mean_) of the PTV, the variations in the MUs and *D*_mean_ of the PTV were less than 1%. The maximum dose differences due to the grid size of 3 mm were respectively 3% (18.1 cGy) and 1% (9.1 cGy) in the spinal cord at angular increments of 10° and 2°.

**Figure 3:**
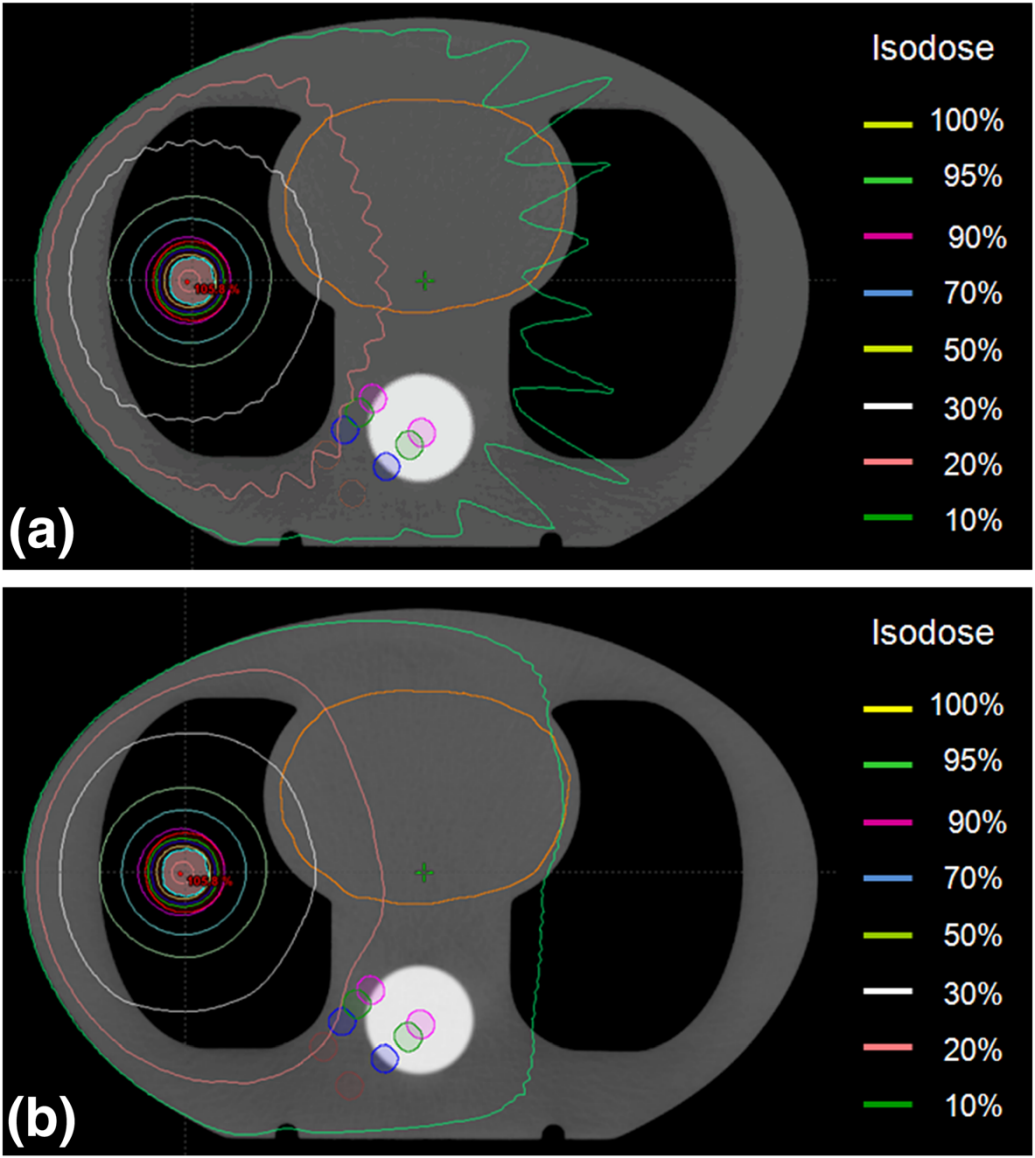
**Undulated dose distributions in an axial plane for the case of *S***_**3**_**.** Doses were predicted for the spherical planning target volume with a diameter of 3 cm using a 2-mm grid size and angular increments of **(a)** 10° and **(b)** 2°.

**Figure 4:**
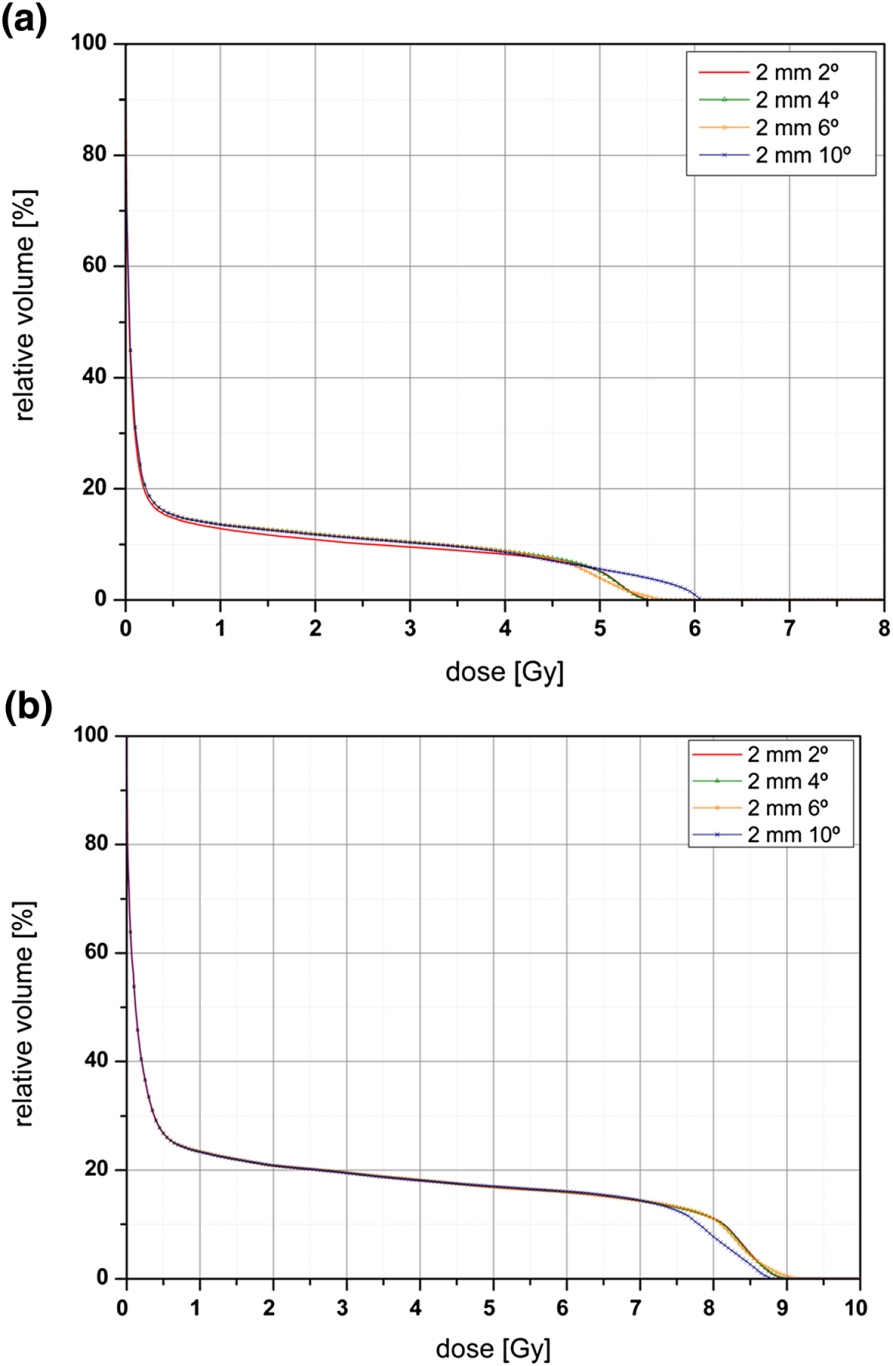
**Dose–volume histograms for the spinal cord at *V***_**11,0**_**.** The volume is located at 11 cm-distance from the isocenter at a 0° azimuthal angle. Histograms are drawn according to the applied angular increments (2°, 4°, 6°, and 10°) in the spherical planning target volume for **(a)***S*_2_ (2-cm diameter) and **(b)***S*_4_ (4-cm diameter).

Dose undulation was observed in the calculated dose distributions, particularly in low-dose regions. The dose distribution estimated using 10° increments showed an undulated isodose pattern at isodose levels lower than 30% for *S*_3_ [Figure 3(a)]. However, an angular increment of 2° provides dose undulation at a 10% isodose level [Figure 3(b)]. Dose undulation generated by discrete calculation using every angular increment of the beam caused over- and under-estimated OAR doses, as shown in DVHs (Figure 4) and listed in Table 2. When a 2-mm grid size was used for dose calculation, an angular increment of 10° predicted a 2% lower (14.5 cGy) and 9% higher (69.1 cGy) maximum dose than a 2° increment in the spinal cord. When the same grid size was used, the large angular increment caused relative dose variation of up to 12%, however, the absolute difference was 9.0 cGy. While the differences for the number of MUs and the PTV dose between a 2° angular increment and larger angular increments increased as the grid size increased in *S*_2_, the angular increment effect on the variation in the number of MUs and the PTV dose value was rarely shown (< 1 cGy) for the large PTV (Table 2).

The plan parameter set of a 3-mm grid size and an angular increment of 4° was able to achieve a dose difference of less than 1% in all OARs tested and in all PTV doses except for a normal lung exposed to a dose of less than 90 cGy in the phantom study for all PTV sizes.

### Patient study

The larger grid sizes caused PTV dose increases of less than 1%, corresponding to average values of 7.3 ± 3.7 cGy for a 3-mm grid and 26.2 ± 6.7 cGy for a 4-mm grid. The increases in the number of MUs were less than 1% (5.9 ± 2.3) and 2% (19.8 ± 5.2), respectively. The variations in the number of MUs and of the PTV and OARs doses are shown in Table 3. However, the number of MUs and the PTV doses were presented according to only the grid sizes by averaging each value at different angular increments, because the angular increment effect on the variation in the number of MUs (<1) and on the PTV doses (< 1 cGy) was insignificant.

**Table 3:** **Monitor units (MUs) and structure doses in the form of a ratio normalized to a reference value from the case of a 2-mm grid size and 2° angular increment, when different plan parameter sets (grid sizes of 2 mm, 3 mm, and 4 mm, and angular increments of 2°, 4°, 6°, and 10°) were used in patient groups divided according to the equivalent diameter (d**_**equi**_) **of the planning target volume (PTV)**

**Patient**	**∆Angle/∆Grid**	**MU**	**PTV (D**_**mean**_**)**	**Heart (D**_**max**_**)**				**Normal Lung (D**_**1000cc**_**)**				**Cord (D**_**max**_**)**				**Esophagus (D**_**max**_**)**			
				**2°**	**4°**	**6°**	**10°**	**2°**	**4°**	**6°**	**10°**	**2°**	**4°**	**6°**	**10°**	**2°**	**4°**	**6°**	**10°**
**(a) G**_**1**_^**a**^																			
Proximal case	2 mm	1.00 (1777)^b^	1.00 (50.32)^c^	1.00 (0.21)	1.00	1.00	1.00	1.00 (2.99)	1.00	1.00	1.00	1.00 (18.03)	1.00	1.00	1.00	1.00 (16.01)	1.00	1.00	1.00
	3 mm	1.00	1.00	1.00	1.00	0.99	0.99	1.01	1.01	1.01	1.01	1.00	1.00	1.00	1.00	1.00	1.00	1.00	0.99
	4 mm	1.02	1.01	1.00	1.00	0.99	0.99	1.03	1.03	1.03	1.03	1.01	1.01	1.01	1.01	1.01	1.01	1.01	1.00
Intermediate case	2 mm	1.00 (1725)	1.00 (51.33)	1.00 (11.49)	1.00	1.00	1.02	1.00 (2.00)	1.01	1.00	0.97	1.00 (9.37)	1.00	1.01	1.02	1.00 (11.54)	1.00	1.00	1.03
	3 mm	1.00	1.00	1.00	1.00	1.00	1.02	1.01	1.01	1.01	0.98	1.00	1.00	1.01	1.02	1.00	1.00	1.00	1.03
	4 mm	1.01	1.01	1.01	1.01	1.01	1.02	1.03	1.03	1.03	1.00	1.00	1.00	1.01	1.02	1.01	1.01	1.01	1.03
Distant case	2 mm	1.00 (1767)	1.00 (51.09)	1.00 (27.57)	1.00	1.00	1.00	1.00 (3.61)	1.00	0.99	1.00	1.00 (8.14)	1.00	1.01	1.04	1.00 (10.23)	1.00	1.00	0.99
	3 mm	1.01	1.01	1.00	1.00	1.00	1.00	1.01	1.01	1.01	1.01	1.00	1.01	1.00	1.04	1.00	1.00	1.00	0.99
	4 mm	1.02	1.02	1.02	1.02	1.02	1.02	1.01	1.01	1.01	1.01	1.01	1.01	1.01	1.04	1.01	1.01	1.01	1.00
**(b) G**_**2**_^**d**^																			
Proximal case	2 mm	1.00 (1542)	1.00 (51.25)	1.00 (0.34)	1.00	1.00	1.00	1.00 (9.38)	1.00	1.00	1.00	1.00 (25.31)	1.00	1.00	1.00	1.00 (20.57)	1.00	1.00	1.00
	3 mm	1.00	1.00	1.01	1.01	1.01	1.01	1.00	1.00	1.00	1.00	1.01	1.01	1.01	1.01	1.00	1.00	1.00	1.00
	4 mm	1.01	1.00	1.00	1.00	1.00	1.00	1.00	1.00	1.00	1.00	1.02	1.02	1.02	1.02	1.01	1.01	1.01	1.01
Intermediate case	2 mm	1.00 (1558)	1.00 (51.08)	1.00 (12.05)	1.00	1.00	1.01	1.00 (1.11)	1.00	1.00	1.00	1.00 (11.19)	1.00	1.00	1.02	1.00 (8.56)	1.00	1.01	1.05
	3 mm	1.00	1.00	1.01	1.00	1.00	1.02	1.01	1.01	1.01	1.01	1.00	1.00	1.00	1.02	1.00	1.00	1.01	1.05
	4 mm	1.01	1.01	1.01	1.01	1.01	1.02	1.03	1.03	1.03	1.02	1.02	1.01	1.01	1.02	1.01	1.01	1.02	1.05
Distant case	2 mm	1.00 (1658)	1.00 (50.68)	1.00 (31.91)	1.00	1.00	0.99	1.00 (1.09)	1.00	1.00	1.00	1.00 (5.54)	1.00	1.00	1.04	1.00 (6.66)	1.00	1.00	1.06
	3 mm	1.01	1.00	1.00	1.00	1.00	1.00	1.01	1.01	1.01	1.01	1.00	1.01	1.00	1.04	1.01	1.01	1.00	1.05
	4 mm	1.01	1.01	1.01	1.01	1.01	1.00	1.03	1.03	1.03	1.03	1.01	1.01	1.01	1.05	1.01	1.01	1.01	1.05
**(c) G**_**3**_^**e**^																			
Proximal case	2 mm	1.00 (1625)	1.00 (52.00)	1.00 (20.55)	1.00	1.00	1.00	1.00 (6.75)	1.00	1.00	1.01	1.00 (24.96)	1.00	1.00	1.00	1.00 (14.41)	1.00	1.00	1.00
	3 mm	1.00	1.00	1.00	1.00	1.00	1.00	1.00	1.00	1.00	1.01	1.00	1.00	1.00	1.00	1.00	1.00	1.00	1.00
	4 mm	1.01	1.00	1.01	1.01	1.01	1.01	1.00	1.00	1.00	1.01	1.02	1.02	1.02	1.02	1.00	1.00	1.00	1.00
Intermediate case	2 mm	1.00 (1506)	1.00 (50.94)	1.00 (16.36)	1.00	1.00	1.01	1.00 (3.82)	1.00	1.00	1.00	1.00 (15.93)	1.00	1.00	1.01	1.00 (16.00)	1.00	1.00	1.01
	3 mm	1.00	1.00	1.00	1.00	1.01	1.01	1.01	1.01	1.01	1.01	1.00	1.00	1.00	1.01	1.00	1.00	1.00	1.00
	4 mm	1.01	1.01	1.01	1.01	1.01	1.02	1.01	1.01	1.01	1.01	1.01	1.01	1.01	1.01	1.00	1.00	1.00	1.00
Distant case	2 mm	1.00 (1566)	1.00 (52.07)	1.00 (0.70)	1.00	1.00	1.00	1.00 (6.23)	1.00	1.00	1.01	1.00 (12.61)	1.00	1.01	1.03	1.00 (18.42)	1.00	1.00	1.00
	3 mm	1.00	1.00	0.98	0.98	-0.98	0.98	1.00	1.00	1.00	1.01	1.00	1.00	1.01	1.03	1.00	1.00	1.00	1.00
	4 mm	1.01	1.00	0.98	0.98	-0.98	0.98	1.00	1.00	1.00	1.01	1.00	1.00	1.01	1.03	1.00	1.00	1.00	1.00

a) The patient group meeting the criteria of the PTV size, 3 cm ≤ d_equi_ < 3.5 cm. Each group includes three patients with different spinal cords distances (ds) from the isocenter as follows: proximal (ds < 6 cm), intermediate (6 cm ≤ ds ≤ 10 cm), and distant (10 cm ≤ ds). b) The reference MU value and c) the reference dose (Gy) were presented as absolute values in parentheses. d) The patient group meeting the criteria of the PTV size, 3.5 cm ≤ d_equi_ < 4.5 cm. e) The patient group meeting the criteria of the PTV size, 4.5 cm ≤ d_equi_ < 5.5 cm.

**Figure 5:**
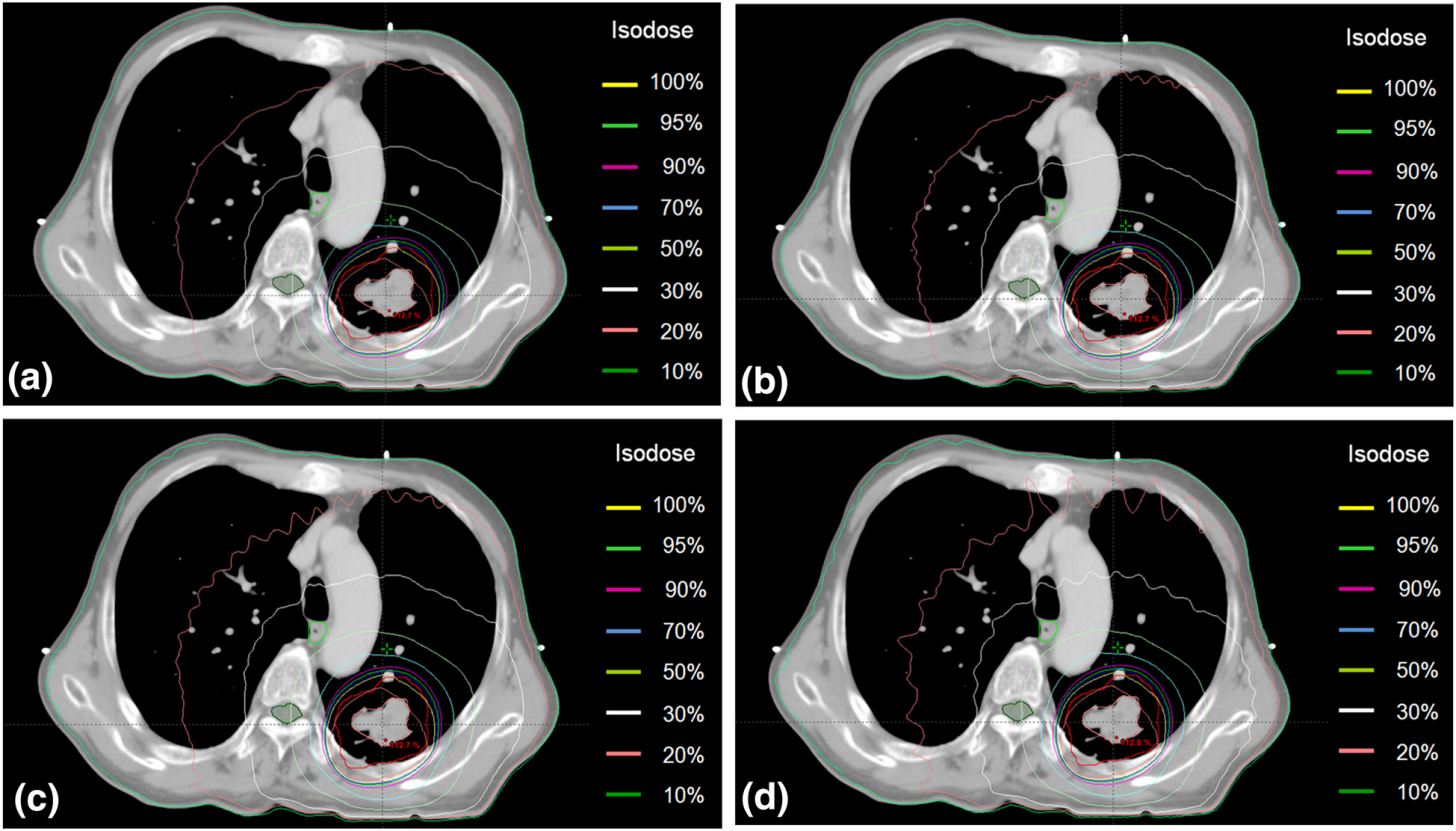
**Comparison of undulated dose distributions for angular increments in the patient cases.** A predicted doses were calculated using a 2-mm grid size and angular increments of **(a)** 2°, **(b)** 4°, **(c)** 6°, and **(d)** 10°.

Although the smallest angle of 2° was used, the 4-mm grid size caused a dose increase of up to 2% (55.3 cGy) in the heart and 2% (46.5 cGy) in the spinal cord. The largest dose variation caused by the 3-mm grid size was less than 1% (21.2 cGy) in the spinal cord, as the same angular increment was used (i.e., a 2-mm grid with a 10° increment vs. a 3-mm grid with a 10° increment). OAR doses were also influenced by the organ position with respect to the isocenter and the beam geometry, as can be seen in the variation in the isodose lines according to the angular increment (Figure 5). While the angular increment of 10° caused the dose difference of 4% (38.4 cGy) to the spinal cord for the distance case (G_1_), the angular increment effect only had a slight effect (< 5.0 cGy) on the spinal cord located relatively close to the isocenter, when the 2-mm grid size was used. The esophagus showed a dose difference from less than 1% to 5% (45.8 cGy) under the larger angular increment, whereas the dose variation in the normal lung dose was less than 5 cGy. When the grid size and angular increment were respectively smaller than 3 mm and 6°, the OAR dose was predicted to have a dose difference of less than 10 cGy (1%) except for the heart, which is exposed to less than 70 cGy.

### Calculation time

We were able to reduce the calculation time by averages of 53 ± 1% and 68 ± 1% in the phantom study and 57 ± 3% and 73 ± 2% in the patient study by using the 3-mm and 4-mm grid sizes (Table 4), respectively, when the same angular increment was used. Using the angular increments of 4°, 6°, and 10° reduced the calculation time by 49 ± 5%, 65 ± 3%, and 78 ± 2% in the phantom study and 48 ± 3%, 64 ± 3%, and 78 ± 2% in the patient study, respectively, when the same grid size was used. With regard to using an appropriate set of grid size and angular increment parameters in the phantom and patient studies, a 3-mm grid size and a 4° (6°) increment could provide dose distributions in a time that is reduced by 78% (85%) with respect to that of the reference set of a 2-mm grid and a 2° increment.

**Table 4:** Comparison of the dose computation time (in the form of a ratio normalized to the time for the case of a 2-mm grid size and a 2° angular increment) in the phantom and patient cases for different plan parameter sets

**Plan parameters**		**Phantom**			**Patients**								
					**G**_**1**_			**G**_**2**_			**G**_**3**_		
					**(3 cm ≤ d**_**equi**_^**a**^ **< 3.5 cm)**			**(3.5 cm ≤ d**_**equi**_ **< 4.5 cm),**			**(4.5 cm ≤ d**_**equi**_ **≤ 5.5 cm)**		
**∆Grid**	**∆Angle**	**S**_**2**_^**b**^	**S**_**3**_	**S**_**4**_	**P**_**1**_	**P**_**2**_	**P**_**3**_	**P**_**4**_	**P**_**5**_	**P**_**6**_	**P**_**7**_	**P**_**8**_	**P**_**9**_
2 mm	2°	1.00	1.00	1.00	1.00	1.00	1.00	1.00	1.00	1.00	1.00	1.00	1.00
		(08 min 45 s)	(09 min 17 s)	(10 min 04 s)	(09 min 45 s)	(11 min 33 s)	(09 min 59 s)	(13 min 44 s)	(10 min 49 s)	(10 min 26 s)	(10 min 34 s)	(11 min 54 s)	(12 min 23 s)
	4°	0.51	0.52	0.50	0.51	0.51	0.55	0.51	0.51	0.51	0.50	0.51	0.51
	6°	0.36	0.35	0.34	0.34	0.34	0.42	0.36	0.34	0.35	0.34	0.37	0.34
	10°	0.22	0.21	0.21	0.20	0.21	0.26	0.21	0.20	0.22	0.21	0.22	0.21
3 mm	2°	0.45	0.47	0.46	0.44	0.42	0.44	0.41	0.44	0.35	0.42	0.43	0.42
	4°	0.23	0.24	0.25	0.23	0.21	0.23	0.21	0.22	0.22	0.21	0.21	0.22
	6°	0.16	0.16	0.17	0.15	0.16	0.15	0.15	0.14	0.16	0.14	0.16	0.14
	10°	0.10	0.11	0.10	0.09	0.10	0.10	0.08	0.10	0.10	0.10	0.10	0.09
4 mm	2°	0.33	0.32	0.31	0.28	0.26	0.27	0.22	0.27	0.29	0.27	0.28	0.26
	4°	0.16	0.16	0.17	0.14	0.13	0.14	0.13	0.16	0.14	0.15	0.14	0.13
	6°	0.11	0.11	0.11	0.10	0.10	0.09	0.10	0.09	0.10	0.09	0.10	0.09
	10°	0.07	0.07	0.07	0.06	0.06	0.06	0.06	0.06	0.06	0.06	0.06	0.05

a) The equivalent diameter of a sphere having the same volume as the planning target volume of patient cases b) Spherical targets of different sizes [S_2_ (2-cm diameter), S_3_ (3-cm diameter), and S_4_ (4-cm diameter)].

## Discussion and conclusions

The interpolated doses based on the coarser grid points generally predicted lower doses than those predicted based on finer grid points in high-dose gradient regions of the generally prescribed isodose level (e.g., 60 to 90%) [1,11]. In addition, the beam penumbra region shows a steeper dose fall-off due to lateral electron disequilibrium, when the small fields are irradiated [22]. Because underdosage around the PTV periphery becomes severe since the effect of electron disequilibrium is critical when using small fields, the dose error in the dose distribution predicted using a coarser grid would be increased at high-dose gradients. We were able to observe the largest dose increase for the PTV and OARs in the plan for *S*_2_.

Niemierko and Goitein evaluated the accuracy of interpolated doses by using linear interpolation according to the grid size and a Fermi function presenting a one-dimensional high-gradient dose profile for beam penumbra [11]. They also described how large grid sizes showed interpolated doses that were lower than the reference doses around the general prescribed isodose level. A lower isodose level should be selected to deliver a higher dose than the prescribed value to meet the PTV dose and coverage [18]. We were able to compare the prescribed isodose level according to the PTV size in a planning system. Selecting a lower prescribed isodose level owing to the lower dose estimated by using large grid sizes required a larger number of MUs. Unintended overdose might be delivered to the patients. This phenomenon was also explored by Dempsey et al. by showing lower PTV coverage for large grid sizes [23]. The discrete beam arrangement based on the angular increment showed a jagged isodose distribution, which is not expected in actual continuous beam delivery. Dose undulation becomes severe as the interval between beam axes increases with large angular increment [24]. As the irradiated field size is decreased for smaller PTVs, the probability that a small critical organ is located outside of the fields would also increase. The larger angular increment effect on structure doses was shown in the plan for *S*_2_ in our study.

Because the discretized dose calculation based on the dose mesh determined by the grid size and its errors are inevitable in a treatment planning system, evaluation of dose distributions with the predicted dose differences according to the variable plan parameters is useful for guiding appropriate plan parameters to achieve a balance between accuracy and efficiency. The possible dose variation due to the angular increment should be considered for OARs in serial tissue such as the spinal cord to avoid unnecessary complications, as the spinal cord showed the largest dose difference under the large angular increment. Sometimes, the spinal cord dose could be insignificant, but even in those cases, it would be necessary to get more accurate dose information for future use such as for the case of either retreatment or treatment of adjacent regions. As one of the alternative methods for efficient dose computation in a planning system, we can consider applying the small angular increment for critical organs and a relatively large angular increment for normal tissues.

We found that a plan parameter set consisting of a 3-mm grid size and a 4° angular increment is suitable for the phantom study and a 3-mm grid size and a 6° angular increment is suitable for patient cases. A phantom study systemically evaluating the range of probable dose differences under the angular increment effect showed that a dose difference of 3% can occur even for spinal cords at the same distance. We were able to observe that one of the hypothetical spinal cords in the intermediate and distant groups showed a dose difference from the reference value of higher than 3%. This could suggest that the dose difference on a small critical organ that is distant from the isocenter can be higher than the acceptable dose error owing to the slight difference in angular position although the dose distributions were created for patients with structures of similar size and anatomical geometry. The 3-mm grid size and 4° angular increment would be more appropriate for DCAT plans for lung SBRT.

To judge whether a 3-mm grid size and 10° angular increment are applicable only to patients with proximal spinal cords, we evaluated dose distributions in three more patient cases with PTVs of different sizes and proximal spinal cords. All evaluated PTV and OARs doses also showed dose differences of less than 1%. We might consider a 3-mm grid size and a 10° angular increment for patients with all small OARs, such as the spinal cord and esophagus, placed within a 6-cm distance from the isocenter. However, it might be difficult to satisfy the dose constraint of spinal cord in lung SBRT, as the spinal cord gets closer to the isocenter. Dose evaluation of small OAR becomes more critical even though the dose difference by the variable plan parameters is insignificant. If we use the plan parameter set of 3-mm grid size and 10° angular increment in dose calculation for patients with a proximal small OAR in serial tissue type, maximum dose of the OAR should be evaluated.

The use of a larger grid size and angular increment led to a reduction in computation time. Although the dose calculation is required for complex tissue composition in patient studies, the time reduction ratio achieved by applying variable plan parameter sets was similar in the phantom and patient studies. When the large grid size is used in the dose calculation, it was possible to reduce the calculation time by approximately at a rate inverse square of the grid sizes. We were also able to speed up the dose calculation by a factor of the inverse ratio of the number of beams for the larger grid size to the number of beams for the reference case, for a particular angular increment. The appropriate plan parameter set can be efficiently determined based on the correlation of the dose calculation accuracy and the time consumption.

Both DCAT and VMAT calculate and deliver optimal planned doses during gantry rotation based on the discrete beam configuration and dynamic MLC apertures at each angular increment. While DCAT delivers conformal doses using a relatively small number of MUs, a constant dose rate, and MLC apertures corresponding to the projection of the PTV at each angular increment [9], VMAT provided a high dose gradient using intensity modulation through a number of deliverable MLC segments converted from non-uniform fluence optimized to satisfy the dose–volume constraints of primary structures in inverse planning [12,24,25]. Under the case where various dynamic components, such as gantry rotation speed, dose rate, and MLC leaf moving speed, are synchronized, VMAT achieves conformal dose distributions. However, VMAT can have more uncertainty when using an intensity modulation technique, particularly for targets involved in significant respiratory motion, owing to the systematic interplay effect between the target motion and the beam aperture motion, as demonstrated by Berbeco et al. [26]. Such uncertainty is expected to be even larger under hypo-fractionation treatment, which is typical in SBRT. In the current health care system, the higher cost of VMAT compared to DCAT is of concern for both billing and human resource utilization. Thus, DCAT is the first choice in our clinic, and VMAT is used only in situations in which it is very difficult to obtain an acceptable dose distribution with DCAT. Examples of such situations include cases where multiple targets are close together, or when critical organs are located extremely close to the target.

In general, it is not easy to predict dose errors for OARs in advance with variable angular increments [8,27]. The systematic evaluation of the dosimetric effect of plan parameters on normal structures in different positions would provide a reference to estimate the approximate error range in DCAT plans. The analysis of dose variation as a function of plan parameters enabled us to determine an optimal set of plan parameters to achieve a balance between accuracy and efficiency in the planning process. Under the conditions considered in this study, a 3-mm grid size and a 4° angular increment are suggested as an optimal set of planning parameters for routine clinical practice with acceptable time efficiency and without a significant compromise in dose accuracy in DCAT.

## Competing interests

This manuscript has not been published or presented elsewhere in part or in entirety, and is not under consideration by another journal. All study participants provided informed consent, and the study design was approved by the appropriate ethics review boards. All the authors have approved the manuscript and agree with submission to your esteemed journal. There are no conflicts of interest to declare.

## Authors’ contributions

JP, SK, and TS participated in study design. SK and TS contributed to conception and organization of the study. JP drafted manuscript and conducted data analysis. HP and JL performed radiation treatment planning. JP, SK, HP, JL, and TS participated in interpretation of the results. YK collected data and delineated contours of target and critical organs. SK and TS edited the manuscript and all authors have read, reviewed, and approved the final manuscript.

## Authors’ information

SK, YK, and TS are members of ASTRO (American Society for Therapeutic Radiology and Oncology). JP, HP, JL, SK, TS joined AAPM (American Association of Physicists in Medicine). JP, HP, JL, YK, and TS are also members of the KOSTRO (Korean Society of Therapeutic Radiology and Oncology) and KSMP (Korean Society of Medical Physics).

JY, Ph.D. Researcher, Research Institute of Biomedical Engineering.

SK, Ph.D. Professor & Director, Clinical Medical Physics and Residency Program in Dept. of Radiation Oncology.

JL, Ph.D. Research Professor & Medical Physicist in Dept. of Radiation Oncology.

HP, Ph.D.: Student majoring in Medical Physics of Radiation Therapy & Medical Physicist in Dept. of Radiation Oncology.

YK, M.D., Ph.D.: Radiation Oncologist, Specialty-Lung and Head & Neck Cancer.

TS, Ph.D.: Professor, Dept. of Biomedical Engineering.
